# Scanning single-molecule counting system for Eprobe with highly simple and effective approach

**DOI:** 10.1371/journal.pone.0243319

**Published:** 2020-12-15

**Authors:** Takeshi Hanami, Tetsuya Tanabe, Takuya Hanashi, Mitsushiro Yamaguchi, Hidetaka Nakata, Yasumasa Mitani, Yasumasa Kimura, Takahiro Soma, Kengo Usui, Michiko Isobe, Takashi Ogawa, Masayoshi Itoh, Yoshihide Hayashizaki, Seiji Kondo

**Affiliations:** 1 Genetic Diagnosis Technology Unit, RIKEN Center for Integrative Medical Science, Yokohama, Kanagawa, Japan; 2 RIKEN Innovation Center, Wako, Saitama, Japan; 3 Advanced Analysis Technology Dept., Medical Technology R&D Division, Olympus Corporation, Hachioji, Tokyo, Japan; 4 K.K. DNAFORM, Yokohama, Kanagawa, Japan; 5 RIKEN Preventive Medicine and Diagnosis Innovation Program, Wako, Saitama, Japan; University of Helsinki, FINLAND

## Abstract

Here, we report a rapid and ultra-sensitive detection technique for fluorescent molecules called scanning single molecular counting (SSMC). The method uses a fluorescence-based digital measurement system to count single molecules in a solution. In this technique, noise is reduced by conforming the signal shape to the intensity distribution of the excitation light via a circular scan of the confocal region. This simple technique allows the fluorescent molecules to freely diffuse into the solution through the confocal region and be counted one by one and does not require statistical analysis. Using this technique, 28 to 62 aM fluorescent dye was detected through measurement for 600 s. Furthermore, we achieved a good signal-to-noise ratio (S/N = 2326) under the condition of 100 pM target nucleic acid by only mixing a hybridization-sensitive fluorescent probe, called Eprobe, into the target oligonucleotide solution. Combination of SSMC and Eprobe provides a simple, rapid, amplification-free, and high-sensitive target nucleic acid detection system. This method is promising for future applications to detect particularly difficult to design primers for amplification as miRNAs and other short oligo nucleotide biomarkers by only hybridization with high sensitivity.

## Introduction

Genomic mutation and changes in the amount of DNA/RNA in biological samples are closely related to various human diseases such as cancer, Alzheimer’s, and infectious diseases. Quick, highly sensitive, cost effective, and easy procedures are required to evaluate these changes quantitatively [1, 2]. However, when a fluorescence-labelled target molecule is evaluated using the ensemble average of the fluorescence signal with a fluorometer, several problems arise with regard to the detection limit, quickness, and consumption of a large sample volume. Therefore, nucleic acid amplification methods such as real-time PCR are routinely applied for highly sensitive target gene detection [3]. However, these require external references, and variations in PCR efficiency by inhibitors in the sample interfere with quantification. Furthermore, when the change in target concentration is relatively small, the counting resolution is limited because of exponential signal amplification. In recent years, digital PCR methods have been proposed to improve detection accuracy and sensitivity [4–6]. Digital PCR divides the solution into small compartments or droplets and examines the amplified molecules according to the segmented regions. Digitalized on/off signals as the result of amplification from target DNA/RNA are determined by the presence or absence of a target single molecule in the compartments or droplets. This enables an extremely accurate counting resolution for target oligonucleotides. In general, carry-over contaminants of amplicon are one of the major risks of qPCR. Therefore, the combination of dUTP and Uracil DNA glycosylase are applied to suppress the contaminants; however, this is not sufficient to completely remove PCR product molecules from all samples [7]. Moreover, droplet-based amplification risks exposing the DNA-amplified products to open experimental environments, which can cause false-positive results by contamination of the template DNA. Additionally, fractionation of the reaction mixture requires expensive disposable equipment. Digital PCR involves end-point detection unlike real-time PCR, but inhibitor risks, which cause false negatives, may still be problematic [5].

On the other hand, techniques for the detection of a fluorescence signal from a fluorescent dye bound to a targeted single molecule without amplification, have also been developed in recent years. In conventional fluorescence measurements such as fluorescence spectrophotometry, a large portion of the solution is irradiated, and the intensity of the fluorescence emission from the species is detected. However, when the concentration is low, the number of molecules in the irradiated region is also low, and the background signals due to scattering, stray light, and thermal noise become stronger than the signal of the target molecule, making precise measurements difficult. However, when the measurement is restricted to a small portion of the solution, the signal emitted from a single molecule passing through the observation area matches the light intensity distribution of the confocal optical system. Consequently, by analyzing the shape of the signal, it is possible to improve the noise discrimination and thus, the accuracy of the measurement [8].

Conversely, other methods, which use confocal optical systems, such as fluorescence correlation spectroscopy (FCS) [9, 10], fluorescence intensity distribution analysis (FIDA) [11], and photon counting histograms (PCH) [12], are also widely used for highly sensitive fluorescence detection of molecules in solution. These methods provide highly accurate results through statistical analysis on a single-molecule level and are based on the photometry of fluorescence emitted from excited fluorescent dyes within the confocal volume of an aqueous solution. Among these techniques, FIDA, which is based on optical scanning, excels in providing extremely accurate single-molecule measurements in solution [13, 14]. The experimental setup for FIDA measurements is the same as that used for optical scanning and detection of molecules. These methods facilitate the detection and analysis of molecules at concentrations as low as 1 nM; however, in the case of even lower concentrations, the frequency at which molecules enter the confocal volume decreases, and the molecular signal is lost in the process of statistical analysis, resulting in detection difficulties.

Detection of molecules in solution without recourse to statistical methods was previously attempted by counting specific photon bursts when fluorescent molecules passed through the confocal volume [15–18]. Ultrasensitive detection has also been realized by flow channels [19–23] and rotating containers [24]. To increase the sensitivity, the counting of high-brightness nanoparticles [25, 26] and oligonucleotide arrays [27] constructed from the target nucleic acids have been reported. These methods can be used to detect molecules modified with multiple fluorophores or large particles, but they experience difficulty attaining sensitivity beyond the femtomolar level for a single fluorescent molecule.

Here, we discriminate the noise from signals derived from a single molecule according to the acquired signal shape as fluorescent molecules, which match the intensity distribution of the exciting light, pass through the confocal volume. A particle detection method based on this kind of signal shape analysis has already been proposed by Altamore et al. [24] for large particles or cells by using a rotating container. However, because of the intense fluctuation of the signal, their method cannot detect single fluorescent dye molecules. We discovered that it is possible to accurately discriminate even a weak signal, such as that from a single fluorescent dye, if the solution is optically scanned while suppressing the background fluctuations.

A previously reported scanning single molecular counting (SSMC) measurement method showed that it is possible to obtain information on the translation and rotation diffusion of fluorescent molecules by combining fluorescence polarization measurement and periodic optical beam scanning [28]. In this paper, we present an example of single fluorescent molecule detection and demonstrate its practical application by showing nucleic acid detection without amplification. Each fluorescent molecule in the solution is counted one by one to determine whether a single molecule is present in the about 40 fL virtually partitioned volume made by confocal optics. The number of partitions used to divide the confocal volume determines the sensitivity of the digital measurements. By contrast, in the case of SSMC, the size of the confocal volume corresponds to the size of the container, and the number of containers corresponds to the measurement time. Consequently, it is possible to select the required measurement accuracy according to the concentration of the substance to be measured. In addition, simple measurements can be conducted without distributing the sample into small containers to compartmentalize single molecules [4–6]. Furthermore, because optical detection is used in the confocal optical system, a wide range of molecular species which can be fluorescently labelled, such as proteins and nucleic acids, can be detected. The detection of a variety of molecular species using a common technique is important in order to reduce the cost of examining certain molecular species in a small number of diagnostics.

The ultrasensitive assay for single molecule detection has great advantages but is less resistant to false positive events under the conditions of very low sample concentrations caused by unbound fluorophore-labelled probe and fluorescent impurities. Therefore, it is necessary to remove them by washing or similar procedures, which is key to the stability of the measurement and increases the number of operations. However, it is expected that this can be suppressed in the case of a fluorophore-labelled probe in which the fluorescence intensity is greatly enhanced by hybridization with the target. Eprobe is an exciton-controlled hybridization-sensitive fluorescent oligonucleotide, which requires only a single labeled position with two covalently attached dye moieties such as thiazole oranges for signal generation [29–32]. The Eprobe fluorescence signal is strongly suppressed by excitonic interactions between the two dye moieties on a single strand. When Eprobe hybridizes to a complementary oligonucleotide, the excitonic interaction between the dyes is disrupted due to the intercalation of thiazole orange to a double strand, and a strong fluorescence signal from the dye moieties appears. We also report direct target oligonucleotide detection by only hybridization using Eprobe without amplification or an enzymatic reaction.

## Methods

### Detection device

**Fig 1:**
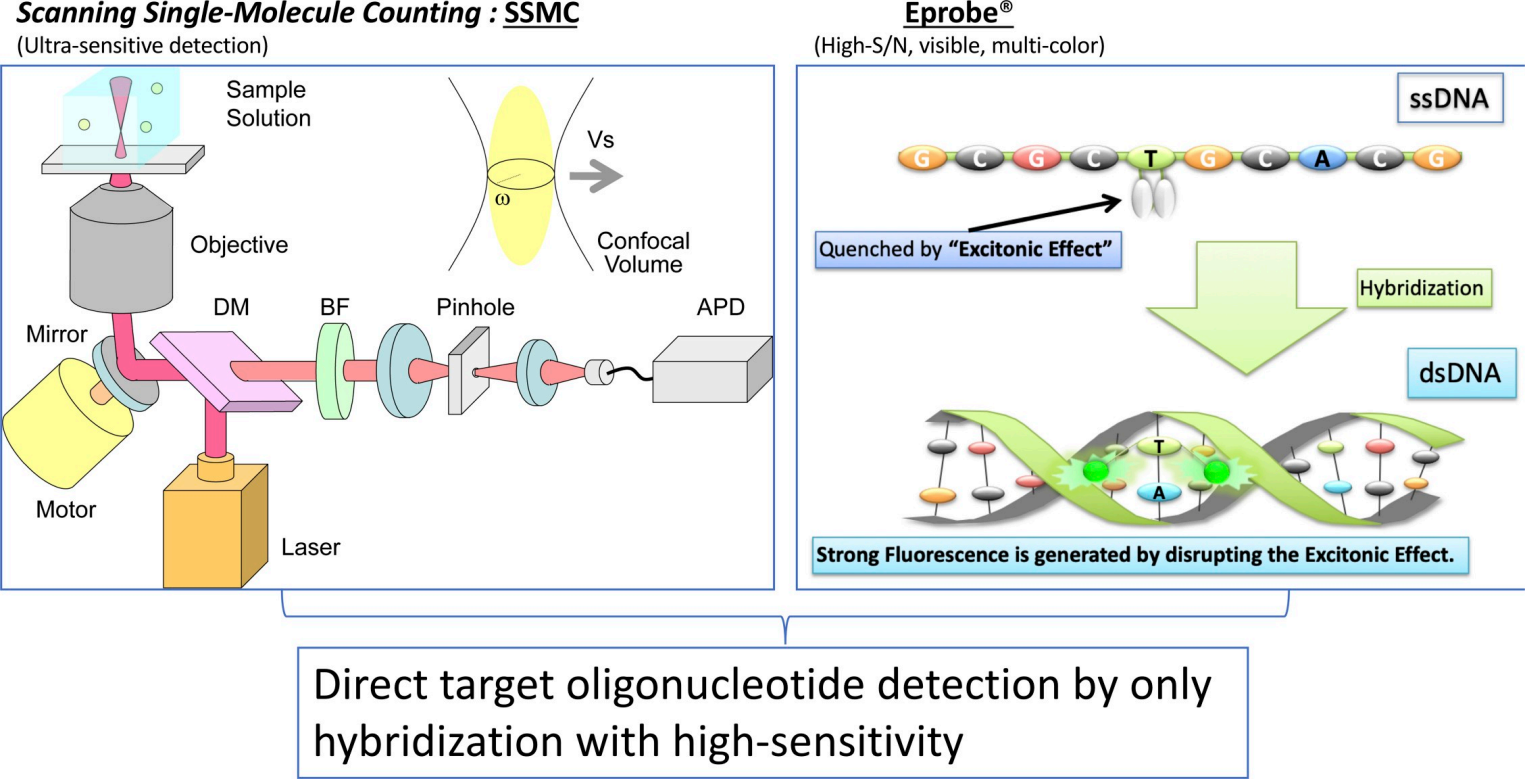
Schematic of optical system used for measurements. Fluorescence intensity is detected as a photon pulse series when the fluorescent dye molecules traverse the confocal volume scanned in the sample solution.

As depicted in Fig 1, the main components of the optical system were a laser light source (Showa Optronics, Yokohama, Kanagawa, Japan) with a wavelength of 642 nm, a dichroic mirror (Chroma Technology Japan, Yokohama, Kanagawa, Japan), a barrier filter transmitting 660 to 710 nm (Chroma Technology Japan, Yokohama, Kanagawa, Japan), a 40× water immersion objective lens (UAPON40XW340, NA = 1.15) (OLYMPUS, Tokyo, Japan), an inclined mirror with a motor placed just before the objective lens for circular scanning of the confocal volume over a radius of 80 μm on the focal plane, and an avalanche photodiode (APD) as the light detector to perform photon counting (PerkinElmer Japan, Yokohama, Kanagawa, Japan). The container holding the aqueous solution used for measurements was a Nunc Lab-Tek chambered cover-glass (Thermo Fisher Scientific K.K., Tokyo, Japan) or a glass-bottom microplate (OLYMPUS, Tokyo, Japan). Photons emitted from the fluorescent dye molecules were incident to the APD, and a corresponding pulse was output from the APD. This was counted using a pulse counter (SSU, Tokyo, Japan) with a bin time of 10 μs and obtained as the photon time series data. The size of the confocal volume was set at approximately 100 fL, which is equivalent to an effective radius of approximately 1.8 μm at the focal plane of the objective lens. The scanning speed of the confocal volume was set to over 10 mm/s, which is faster than the Brownian motion speed of the molecules. Thus, the evolution of the light intensity time series data reflected the confocal volume intensity profile, which clearly showed the crossing of single molecules.

### SSMC analysis methods

**Fig 2:**
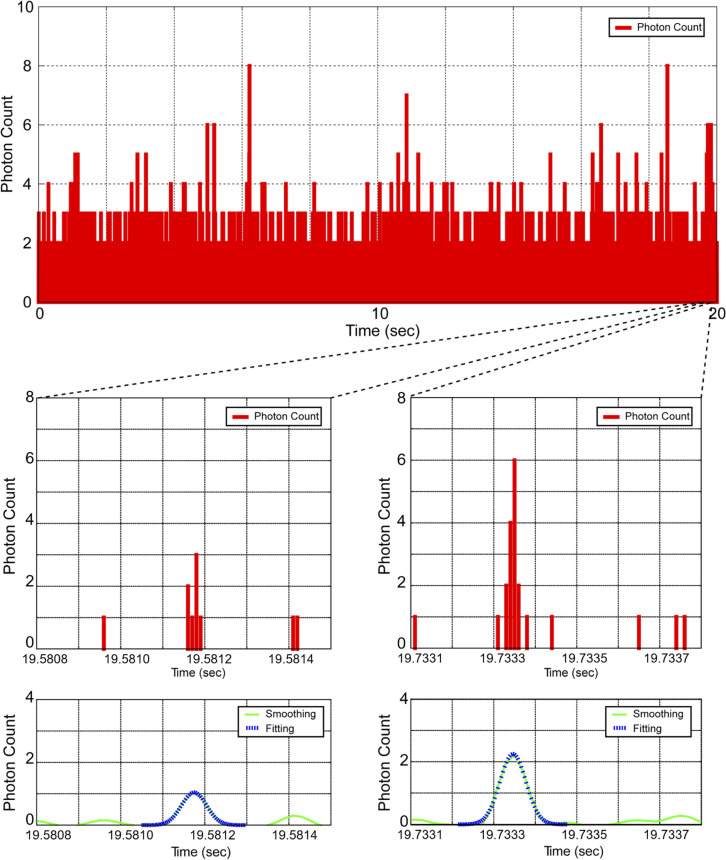
Raw signals of photon time series data. A typical photon pulse sequence for an aqueous solution of ATTO 647N scanned at a linear scanning speed of 77 mm/s (top). A photon pulse series showing the passage of a single fluorescent dye molecule (bottom).

As illustrated in Fig 2, we performed smoothing of the photon time series data (10 μs each) based on the Savitzky–Golay method [33] with a 110 μs window size and five repetitions before analysis. These parameters were determined as S/N increased (S1 Fig).

The smoothed time series data (*I*(*t*)) were differentiated and the convex region of the signal was extracted and fitted using the Levenberg–Marquardt method for each item to a Gaussian function (Eq (1)).

$$I(t)=Aexp\left(-\frac{{\left(t-t_{cen}\right)}^{2}}{2c^{2}}\right),$$ (1)

where *A* is the peak intensity of the Gaussian function, *t*_cen_ is the center time of the peak, and $c=fwhm/(2\sqrt{2ln2})$.

In regions with a correlation coefficient of 0.9 or more, *A* > 1 photon/10 μs, and the region with a FWHM of 20–300 μs is considered to be where the change in signal is consistent with the intensity distribution of the excitation light and is counted as the signal of the fluorescent molecule.

For evaluation of the SSMC analysis performance, we applied the conventional method for acquiring the fluorescence intensity determined by the number of photons per unit time using the same device.

### Measurement of dye concentration

A 1 pM ATTO 647N solution (ATTO-TEC GmbH, Siegen, Germany) was prepared in Buffer A (1 mM Tris-HCl, pH 8.0, 0.01% Triton X-100 [Sigma-Aldrich Japan, Tokyo, Japan]). Twenty-one steps of a quarter-logarithmic dilution series (1.78-fold for each steps) were prepared with Buffer A for concentrations ranging from 1 pM to 10 aM.

For measurements, optical scanning was performed at a scanning speed of 77 mm/s, excitation light (642 nm, 1 mW) was irradiated at room temperature, and fluorescence time series data were acquired thrice (in all instances) in the range of 0–592 aM for 1200 s, 1–5.62 fM for 200 s, 10–56.2 fM for 80 s, 100–562 fM for 40 s, and 1 pM for 20 s.

### Measurement of target oligonucleotide using Eprobe

Eprobe (5′-CTACtACCTCA-3′, t: fluorescence dye labelled position) was purchased from K.K. DNAFORM (Yokohama, Kanagawa, Japan), and target DNA oligonucleotide (5′-TGAGGTAGTAGGTTGTATAGTT-3′) was purchased from FASMAC (Atsugi, Kanagawa, Japan). The Eprobe was dissolved in Buffer B (10 mM Tris-HCl, pH 8.0, 400 mM NaCl, 0.05% pluronic F-127 [Sigma-Aldrich Japan, Tokyo, Japan]) at 100 pM, to which 100 pM target oligonucleotide was added to prepare the hybridization solution. Denaturation and annealing were carried out at 95°C for 1 min followed by cooling at 45°C. The solutions were analyzed under the following conditions that were different from the dye measurement: laser light source of 488 nm, 10 mW (Melles Griot, Thebarton, SA, Australia), and barrier filter transmission of 600–660 nm (Chroma Technology Japan, Yokohama, Kanagawa, Japan). The measurement time was 600 s.

## Results

### Evaluation of single molecule detection by scanning of confocal region

To evaluate the single molecule detection by SSMC analysis, we verified the correlation between the scanning speed of the confocal region and width of the pulse series. When a molecule passes through the confocal volume, the shape of the photon counts and the time series correspond to a Gaussian function representing the intensity distribution of the excitation light.

**Fig 3:**
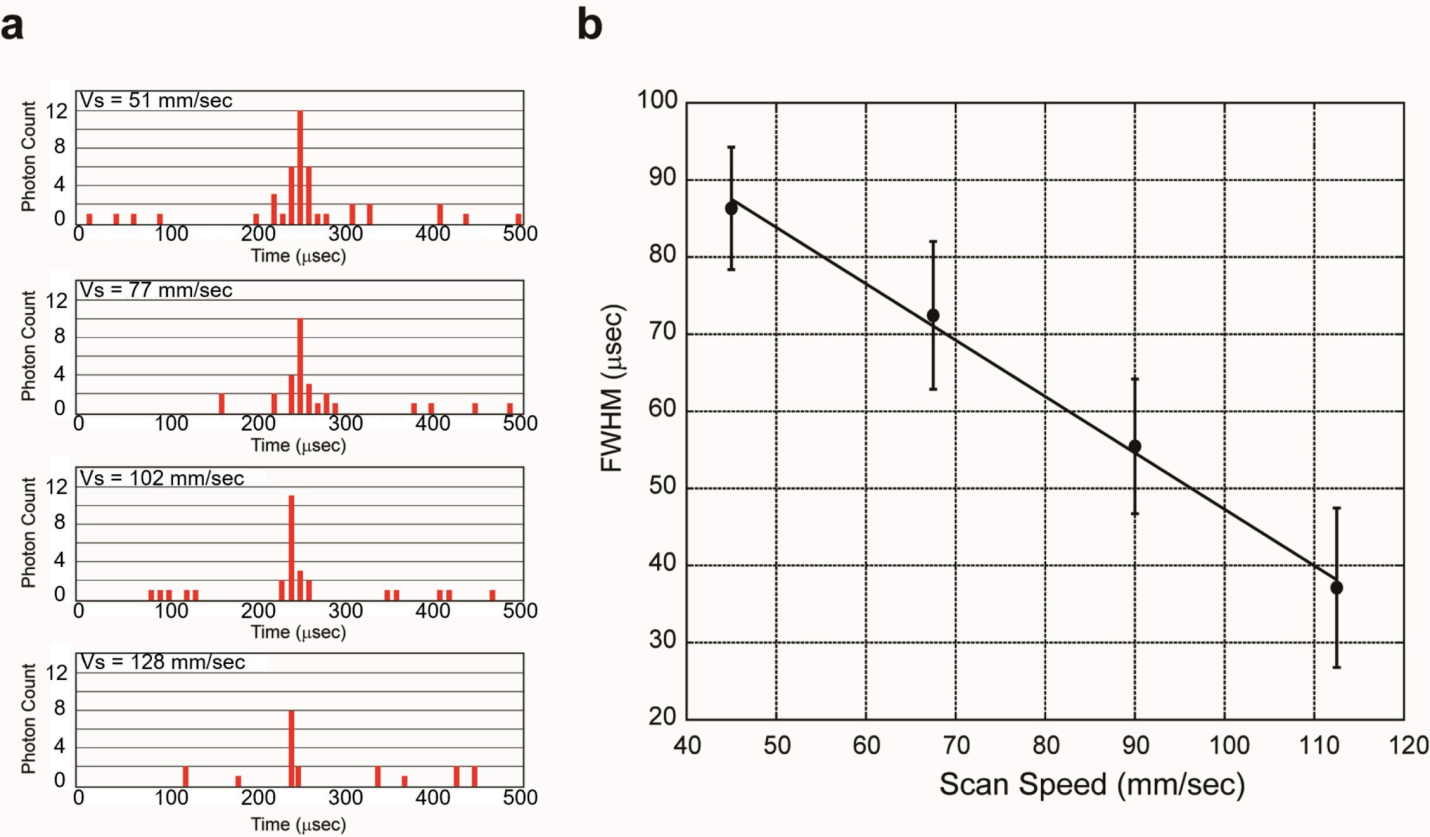
Evaluation of single molecule detection by scanning of confocal region. (A) Typical photon pulse series obtained by scanning a 1 pM ATTO 647N solution. From top to bottom, the speeds are 51 mm/s, 77 mm/s, 102 mm/s, and 128 mm/s, respectively. (B) Relationship between scanning speed and FWHM of signal pulse. Measuring 1 pM ATTO 647N solution at scanning speeds (*V*_s_) of 45 to 110 mm/s. The average FWHM of signals fitting the Gaussian function was plotted. Error bars are the SD calculated from around 100,000 peaks of three measurements.

Photons emitted from the fluorescent molecules were detected with an APD, counted by integrating the photons every 10 μs (a bin time of 10 μs is shorter than the time required for the molecule to pass through the confocal region), and the time series data of the photons were collected. Fig 3A shows an example of a typical photon pulse sequence generated when fluorescent dye molecules traverse the confocal volume. This is a fraction of the total measurement time in seconds per unit and represents raw data displaying the number of photons detected every 10 μs of bin time.

Two types of cases were present: cases in which continuum photon pulses were observed (Fig 3A, around 250 μs), and cases in which they were observed discretely (Fig 3A, around 0–200 μs and 300–500 μs). By modifying the scanning speed of the confocal volume, we confirmed that the width of the pulse series changes in accordance with the scanning speed (Fig 3B). Further, the full-width at half-maximum (FWHM) was found to be inversely proportional to the scanning speed. This result shows that the device can detect a single molecule with these scanning speeds in the SSMC analysis system. The noise reduction was optimized using the smoothing width and repeating time in the Savitzky–Golay method. When the smoothing width and repeating times increase than this value, the background peaks in the absence of ATTO 647N in the solution are largely reduced, but the number of observed peaks in the presence of ATTO 647N in the solution was also slightly decreased. However, excess of smoothing width and repeating times decrease necessary fluorescence peaks. From these results, a smoothing width of 110 μs and 5 repeat times were appropriate for noise reduction, as shown in S2 Fig.

### Comparison of detection performance

**Fig 4:**
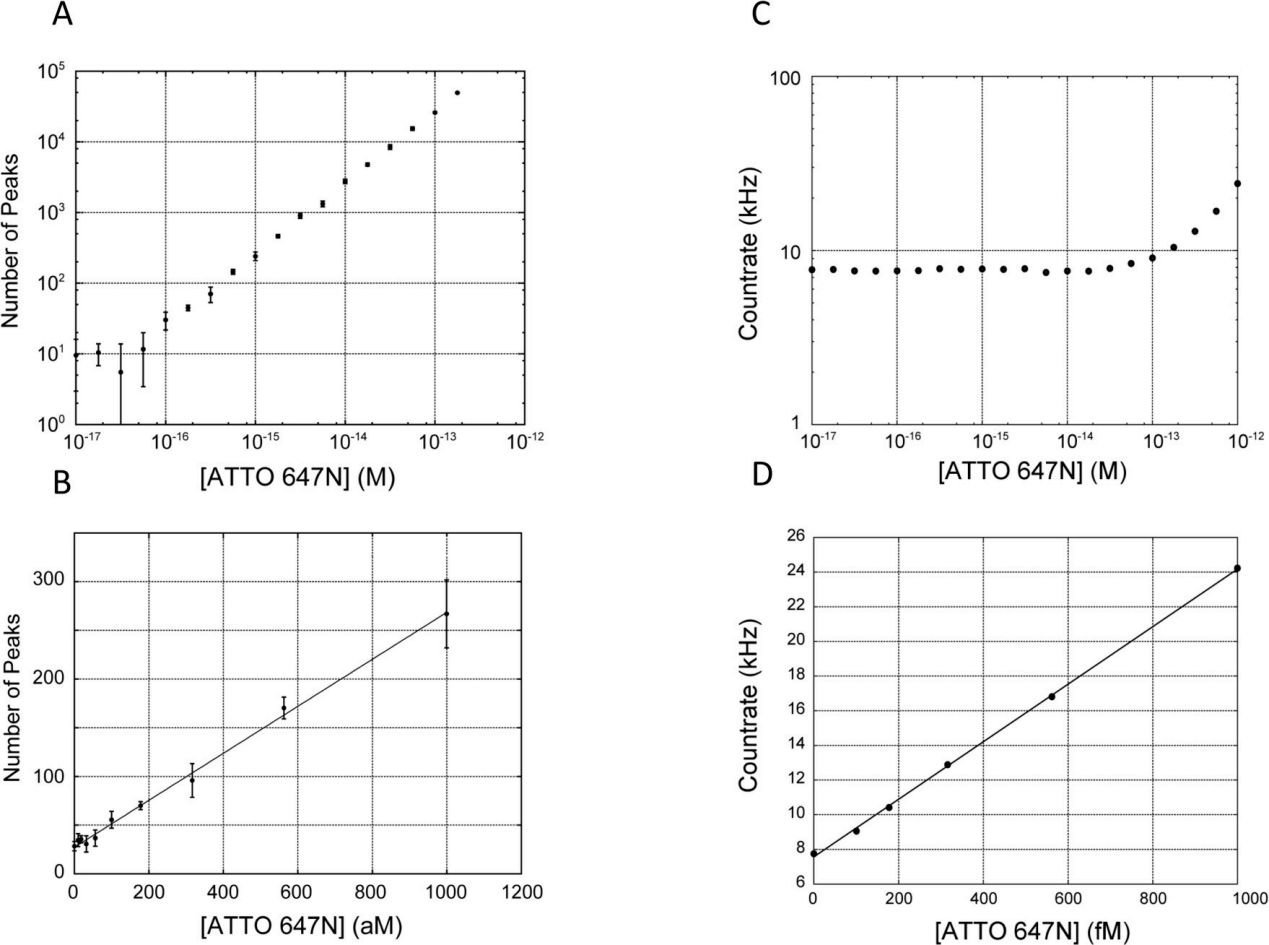
Comparison of LOD between conventional methods and SSMC analysis. (A) Log-log plot of ATTO 647N measurement by SSMC method. Raw traces of photon count for each concentration are shown as S3 Fig. (B) Plot for 0 to 1000 aM ATTO 647N. The number of peaks detected was converted for a measurement time of 600 s. Error bars are the SD of three measurements. The detection limit was 62 aM based on 3SD of the background. (Linear fit *y* = 0.24*x* + 28, *R*^2^ = 0.996 by SSMC analysis) (C) Log-log plot of the dye concentration in the range of ATTO 647N 10^−17^ to 10^−12^ and the countrate. (D) Plots in the range of ATTO 647N 0 to 1 pM (*y* = 16.6*x* + 7.55, R2 = 0.995) and the detection limit was 12 fM.

To determine the limit of detection (LOD) and dynamic range of SSMC analysis, we tested a serial dilution range of 0 to 1000 fM ATTO 647N solutions by acquisition of the photon time series data (Fig 4). It is necessary to observe the molecules within a short time as they pass through the confocal optical system in SSMC. Therefore, because the ATTO 647N dye is highly resistant to photobleaching and its triplet quantum yield is low, it was deemed suitable for SSMC.

The number of peaks was plotted as a function of the dye concentration. On the basis of the slope of the straight line, a linear approximation of the number of molecules detected indicated a detection capacity of 24 peaks/100 aM (6.0 ×10^10^ molecules/L)/10 min (Fig 4B).

In SSMC analysis, there is a consistent relationship between the number of molecules (peak number: *N*) and the other parameters—scanning speed (*V*_*S*_), effective scanning sectional area (*S*), measurement time (*T*_*S*_), and concentration of the sample solution (*M*)—as shown in Eq (2).

$$S=\frac{N}{V_{S}T_{S}M}$$ (2)

The effective scanning sectional area is *S* = 9.63 μm^2^ because *V*_s_ = 69 mm/s and *T*_s_ = 600 s. If the sectional area of the confocal volume is circular, the effective radius (*r*) would be *r* = 1.75 μm. This value is consistent with the radius in the lateral direction of the confocal volume, where *ω*_1_ ≈ 1.8 μm, which was assumed from the FCS measurement obtained beforehand using ATTO 647N (S2 Fig). In the case of this confocal region, a count number is calculated for N = 24.8 at 100 aM, so that the count number in SSMC analysis almost coincides with the calculated value. Concentration measurements were carried out by counting the peaks passing through the observation area according to a Gaussian distribution (Fig 2).

We measured the signal-to-noise ratio (S/N) in a 100 fM ATTO 647N solution. The fluorescence intensity measured by SSMC device has S/N = 1.2 as determined from the values of the dye solution (9.1 Hz) and no dye solution (7.7 Hz) as background signal by the value of each photon per unit time. However, after SSMC analysis, the S/N ratio was significantly increased to S/N = 8070.2 as determined from the values of the dye solution (25824.8 counts) and background signal (3.2 counts) by the number of each fluorescent molecule count. SSMC analysis showed a large increase of 8000-fold compared with that of the conventional method despite the concentration being as low as 100 fM.

The LOD by SSMC analysis was determined by extrapolating the ATTO 647N concentration at the number of peaks equal to the background plus three times the standard deviation (SD) of the background. The LOD of SSMC analysis was consequently calculated to be 28–62 aM from two experiments. As 30 μL of test solution was used, 1.9 zmol (about 1100 molecules) could be detected. On the other hand, the LOD by the conventional method for the analysis of number of photons per unit time was 12 fM, and the LOD by counting photon bursts was 2.5 fM (Table 1). The SSMC method achieved 300-fold and 40-fold larger improvement in the LOD of ATTO 647N than each method.

**Table 1:** The Limit of Detection (LOD) of each method.

Method	SSMC analysis (Fig 4A and 4B)	Analyzing photons number per unit time (Fig 4C and 4D)	Counting photon bursts (S4 Fig)
**LOD**	28–62 aM	12 fM	2.5 fM

### Comparison of signal-to-noise ratio of the detection signal from Eprobe

**Fig 5:**
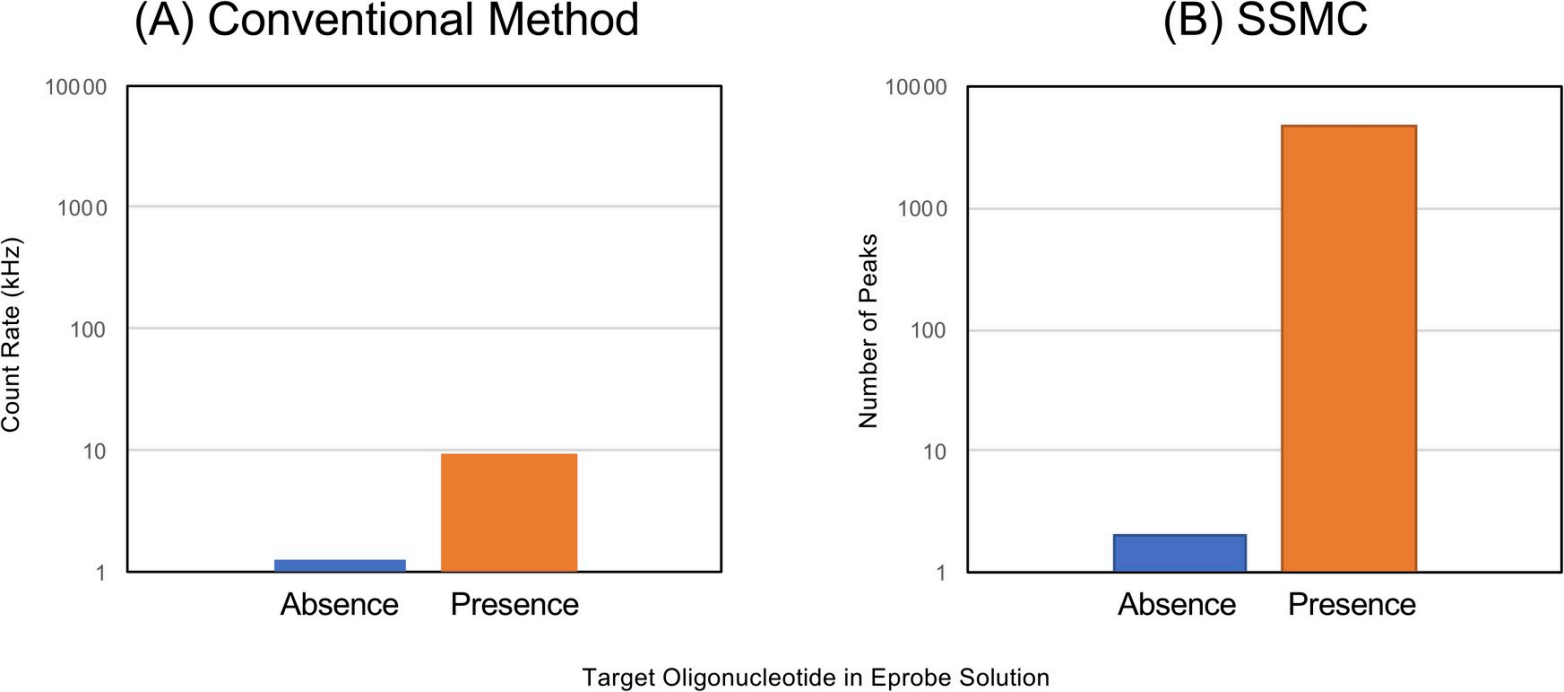
Comparison of signal-to-noise ratio for oligonucleotide detection using Eprobe. The signal values of each method were detected in the absence/presence of target oligonucleotide in 100 pM Eprobe solution. (A) Oligonucleotide detection by conventional method. Count rates in the absence and presence of target oligonucleotide are 1.2 kHz and 9.1 kHz, respectively. (B) Oligonucleotide detection by SSMC. Numbers of peaks in the absence and presence of target oligonucleotide is 2 and 4652, respectively.

To evaluate the performance of the target oligonucleotide detection signal from Eprobe, we measured the S/N of an Eprobe solution in the presence/absence of the target oligonucleotide. We assembled the device for 488 nm excitation and 600–660 nm detection to avoid Raman scattering from water. We conducted a hybridization reaction before measurement in the presence of the target oligonucleotide, and then measured the S/N of a 100 pM Eprobe solution, similar to the ATTO 647N. The fluorescence intensity measured by SSMC device is S/N = 7.6 (Fig 5A). However, after SSMC analysis, the S/N significantly increased to S/N = 2362 (Fig 5B). The SSMC/Eprobe method resulted in a 300-fold improvement in S/N.

## Discussion

Here, we demonstrated a rapid and ultra-sensitive detection method, SSMC, for the quantification of fluorescence signals with a simple procedure and short measurement time (600 s) for 100 aM fluorophore solutions. Using this detection method, we achieved a good S/N for the detection of a target oligonucleotide using a hybridization-sensitive fluorescent probe, Eprobe, without amplification or an enzymatic reaction.

A feature in the peak analysis of the SSMC method is to discriminate the shape of the fluorescence signal. Usually, the weak fluorescence signal from a single molecule causes discrete observation, which makes peak analysis difficult. However, an approximation to a Gaussian function through smoothing allows for the detection of a fluorescence signal from a single molecule. The fact that the signal analyzed in this way reflects the passage of a single molecule suggests that the FWHM changes as a result of changes in the scanning speed (Fig 3B). On the other hand, signals derived from scattering, stray light, and thermal noise are observed with a constant probability without any variation in time, irrelevant to the shape of the intensity distribution of the confocal optical system. It is therefore possible to eliminate noise using an approximation based on the Gaussian function.

In the analysis of the number of photons by simple thresholding per unit time using conventional fluorescence intensity measurements, the best LOD for the dye (ATTO 647N) was 12 fM when the same data for SSMC ATTO 647N measurement in Figs 2 and S4 were utilized with threshold (TH) = 25. In the low concentration region in the simple thresholding method, the linearity of the calibration curve is not maintained due to the influence of the background signal. This can be attributed to the fact that the photons contained in the measured data were derived almost exclusively from scattering, stray light, and thermal noise.

Methods based on counting photon bursts have been proposed for the detection of particles [14, 16]. When this analytical method is adopted to count photon bursts, the number of detectable molecules is reduced to approximately 1/10, and the LOD is only 2.5 fM (S4 Fig). When molecules traverse the bright section of the central portion of the confocal volume, many photons are generated; consequently, even analytical methods based on photon burst counting can detect the molecules. However, when molecules pass through the dark portion of the periphery of the confocal volume, the signal is small and thus miscounting occurs. This reduction in sensitivity is unlikely if the sample consists of large and bright particles or if the molecules have multiple fluorophores.

When SSMC analysis was combined with hybridization-sensitive Eprobe, we achieved a good signal-to-noise ratio (S/N = 2362) between the presence and absence of 100 pM target nucleic acid. Since the S/N of Eprobe is about 10 in the conventional fluorescence measurement method, this combination significantly improved the S/N. This results from the reduction of the background signal from excess Eprobe by the SSMC/Eprobe combination, and the detection limit is expected to be less than several pM. In the case of ATTO 647N, the fluorescence maximum (664 nm) is within the range of the filter (660–710 nm). On the other hand, the detection is performed at a longer wavelength (600–660 nm) than the fluorescence maximum of Eprobe (530 nm), so a very weak signal is detected in order to avoid background signals such as those originating from Raman scattering. However, the sensitivity will greatly increase to the fM range that is necessary to detect nucleic acid biomarkers such as miRNA in blood, if the fluorescence intensity in this region is improved using a fluorescent dye using a large Stokes shift dye or Förster resonance energy transfer technology.

The method is not inhibited by quantification due to amplification bias or false positives due to false amplification in PCR because this system does not amplify the target sequence. The system requires a very simple process of mixing the target oligonucleotide and Eprobe and performing hybridization for sensitive measurements. Although quantification without amplification at the single-molecule level is very intuitive, target binding to the solid phase and washing of the excess fluorescent probe requires longer experimental time for detection due to the many handling processes. SSMC/Eprobe will overcome this issue since our system requires only a hybridization reaction in solution and a short handling time for sample preparation. This simplifies the detection process and reduces the cost for single molecule detection.

Therefore, we aim to apply SSMC analysis to a simple, rapid, amplification-free, and high-sensitive target nucleic acid detection system, which can be achieved by only hybridization via the combination of SSMC and Eprobe. It is expected that non-amplified and digital detection can be achieved for miRNAs, etc., for which it is particularly difficult to design primers for amplification and whose variation as a disease marker is not very large.

## Supporting information

S1 Fig Effects of analysis parameters. Number of peaks and analysis parameters (smoothing width and repeat times). Analysis of 100 s of simulation data including 10000 peaks. Smoothing width of 70–150 μs of Savitzky–Golay method was analyzed with one to nine repetitions.
(TIF)

S2 Fig FCS measurement of 1 pM ATTO 647N. Measurement performed at 642 nm with a 2 mW excitation for 10 s. Autocorrelation function (black), fitting curve by S1 Eq (red).
(TIF)

S3 Fig Raw traces of photon count for measurement of ATTO 647N. Photon count for each concentration (0 M, 100 aM, 1 fM, 10 fM, 100 fM, 1 pM) were shown as Fig 4A. Fluorescent intensity of each concentration as 7.8 kHz (0 M), 7.7 kHz (100 aM), 7.8 kHz (1 fM), 7.7 kHz (10 fM), 9.1 kHz (100 fM), 22.4 kHz (1 pM).
(TIF)

S4 Fig Relationship between number of photon bursts and dye concentration. Data were collected by the same device as in Fig 4. (a) Log-log plot of dye concentration in the range of ATTO 647N 10^−17^ to 10^−12^ and the number of photon bursts with threshold (TH) = 20, 25, 30 counts. (b) Plots in the range of ATTO 647N 0 to 60 fM (*y* = 7.59 *x*, *R*^2^ = 0.977) with TH = 25, where the LOD is 2.5 fM.
(TIF)
