# Supplementary figures and images for: Scanning single-molecule counting system for Eprobe with highly simple and effective approach
Source: PLoS One. 2020 Dec 15;15(12):e0243319. doi: 10.1371/journal.pone.0243319 (PMC7737986; doi:10.1371/journal.pone.0243319)

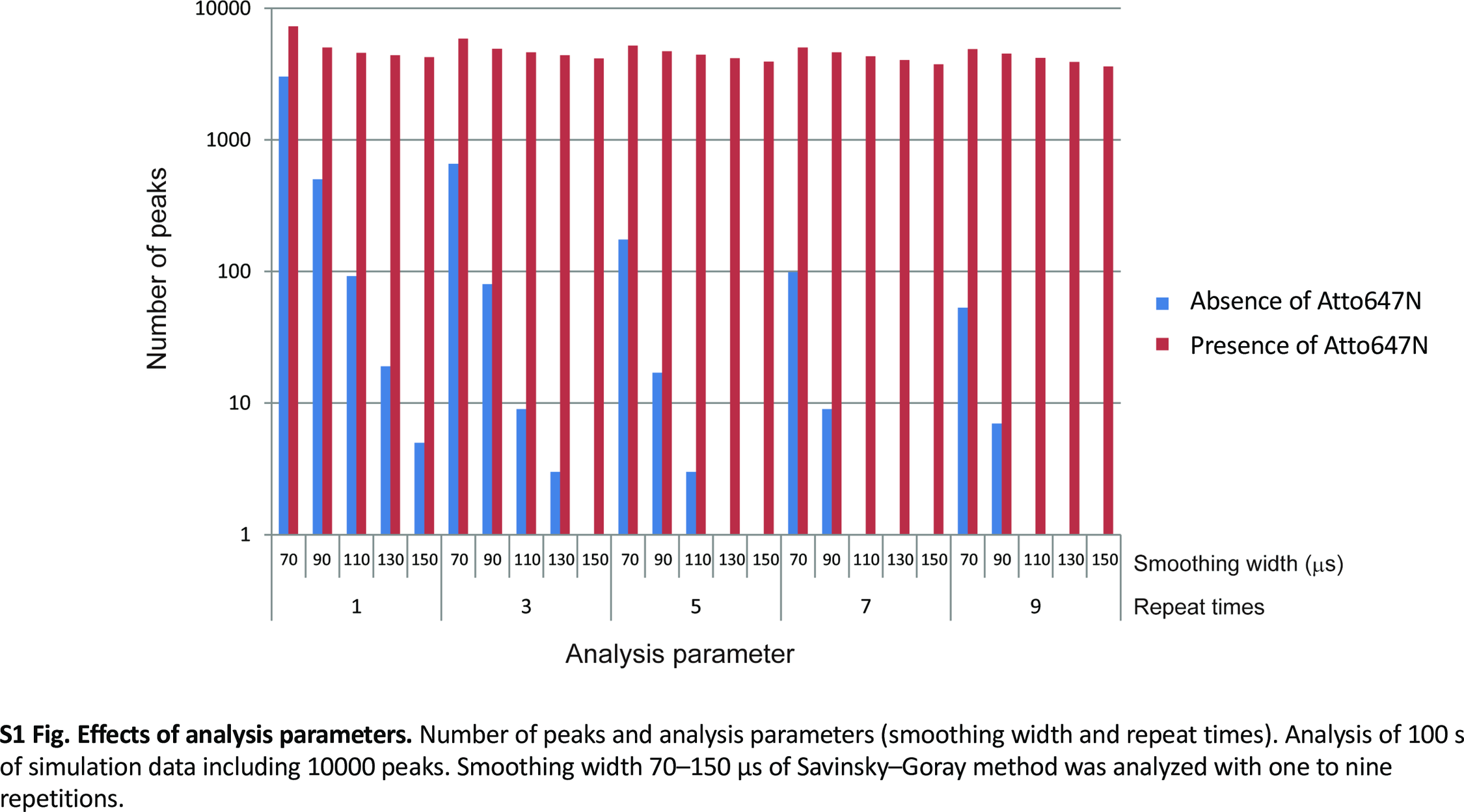

Supplement: S1 Fig — Number of peaks and analysis parameters (smoothing width and repeat times). Analysis of 100 s of simulation data including 10000 peaks. Smoothing width of 70–150 μs of Savitzky–Golay method was analyzed with one to nine repetitions. (TIF) [file pone.0243319.s001.tif]

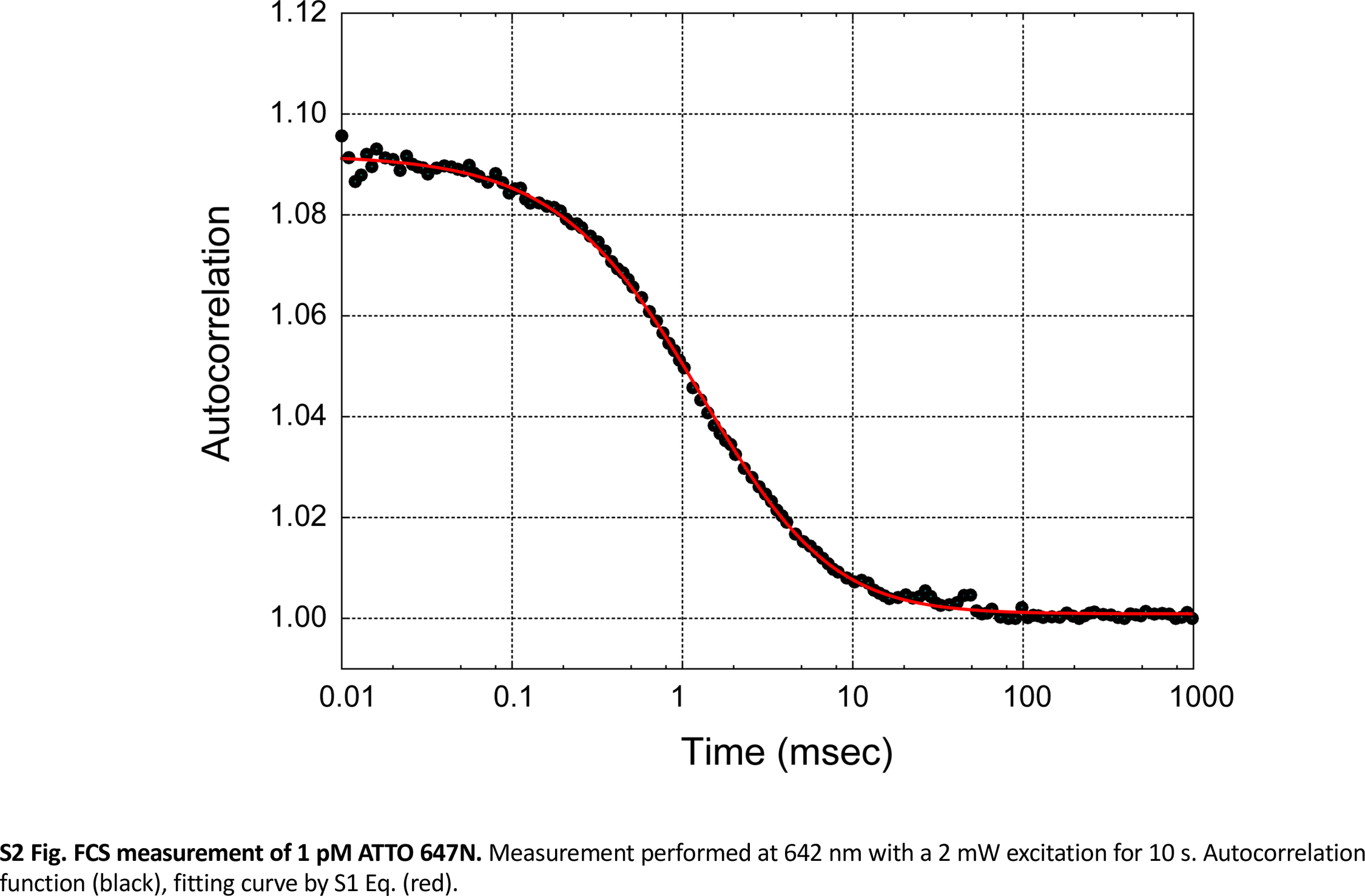

Supplement: S2 Fig — Measurement performed at 642 nm with a 2 mW excitation for 10 s. Autocorrelation function (black), fitting curve by S1 Eq (red). (TIF) [file pone.0243319.s002.tif]

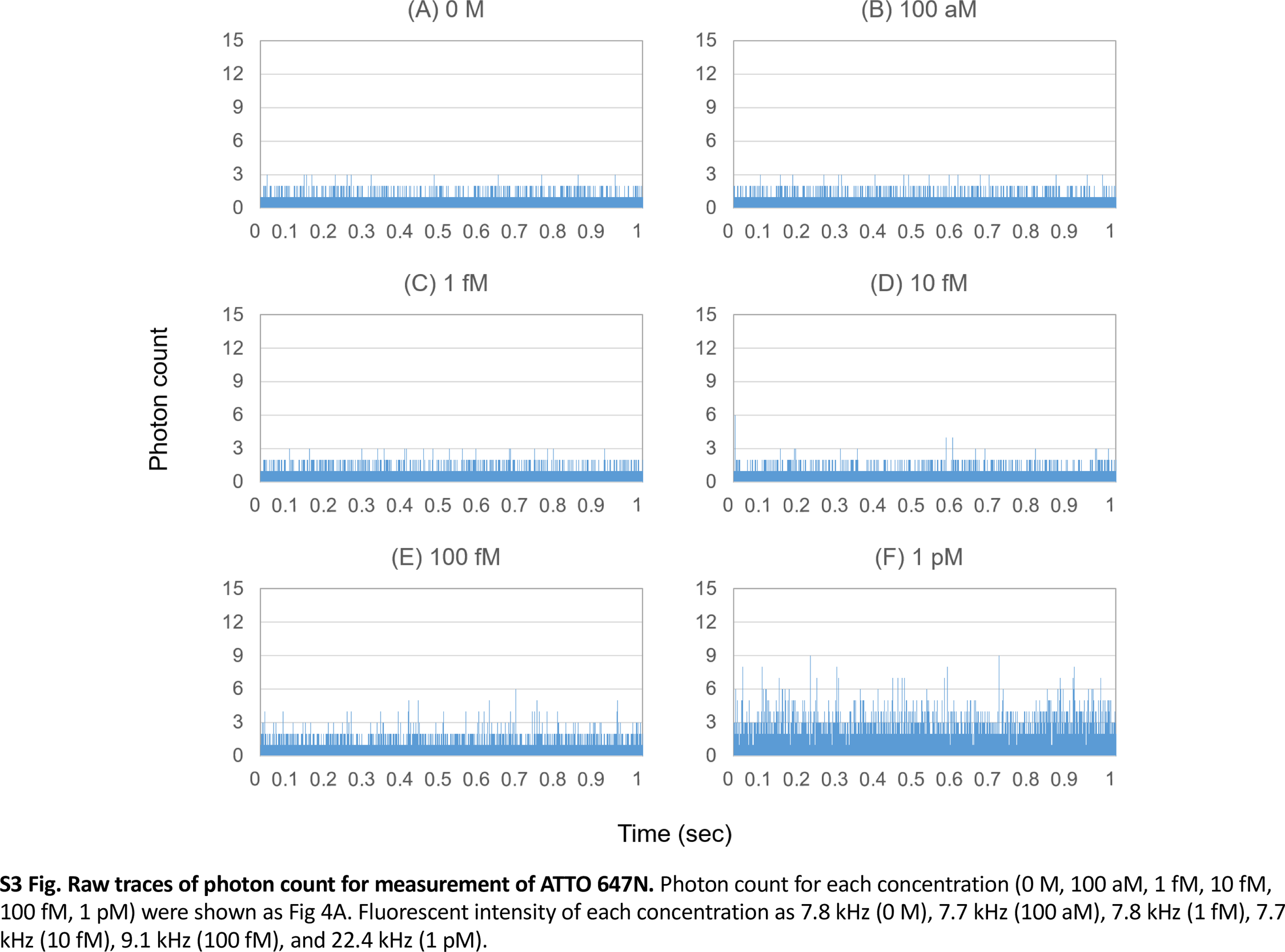

Supplement: S3 Fig — Photon count for each concentration (0 M, 100 aM, 1 fM, 10 fM, 100 fM, 1 pM) were shown as Fig 4A. Fluorescent intensity of each concentration as 7.8 kHz (0 M), 7.7 kHz (100 aM), 7.8 kHz (1 fM), 7.7 kHz (10 fM), 9.1 kHz (100 fM), 22.4 kHz (1 pM). (TIF) [file pone.0243319.s003.tif]

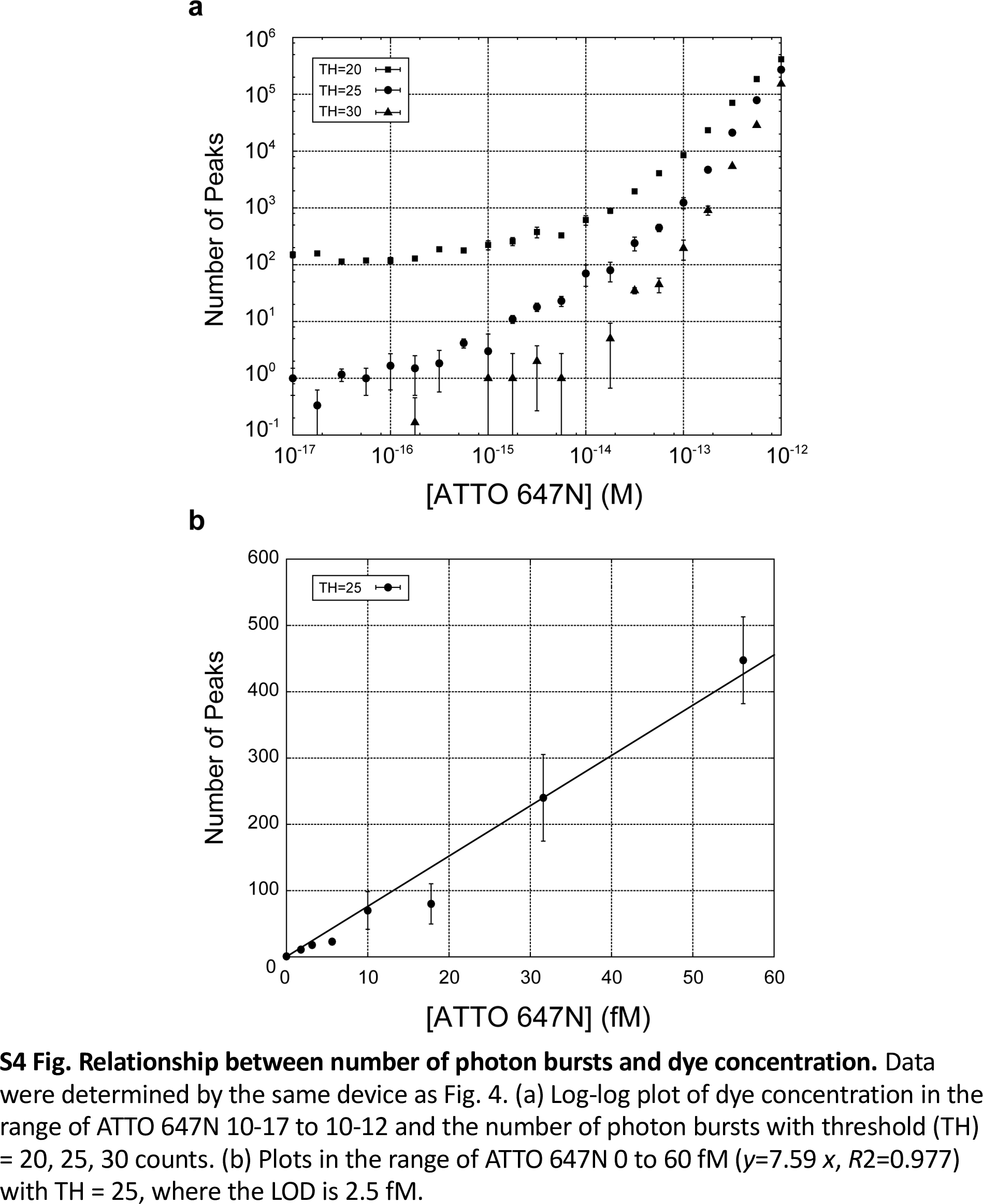

Supplement: S4 Fig — Data were collected by the same device as in Fig 4. (a) Log-log plot of dye concentration in the range of ATTO 647N 10−17 to 10−12 and the number of photon bursts with threshold (TH) = 20, 25, 30 counts. (b) Plots in the range of ATTO 647N 0 to 60 fM (y = 7.59 x, R2 = 0.977) with TH = 25, where the LOD is 2.5 fM. (TIF) [file pone.0243319.s004.tif]
